# Influence factors of extra-articular manifestations in rheumatoid arthritis

**DOI:** 10.1515/med-2020-0217

**Published:** 2020-08-26

**Authors:** Xing Chen, Mingmei Zhang, Tao Wang, Yunming Li, Meng Wei

**Affiliations:** Department of Rheumatology and Immunology, The General Hospital of Western Theater Command, Chengdu, Sichuan Province, 610083, China; School of Medicine, Southwest Jiaotong University, Chengdu, Sichuan Province, 610031, China; Department of Medical Management, Division of Health Services, The General Hospital of Western Theater Command, Chengdu, Sichuan Province, 610083, China; Department of Statistics, College of Mathematics, and School of Medicine, Southwest Jiaotong University, Chengdu, Sichuan Province, 610031, China; School of Public Health, Southwestern Medical University, LuZhou, Sichuan Province, 646000, China

**Keywords:** rheumatoid arthritis, extra-articular manifestations, tissue-specific/systemic EAMs, influential factors

## Abstract

**Background and objective:**

Extra-articular manifestations (EAMs) are serious outcomes or complications of rheumatoid arthritis (RA) with increasing mortality and morbidity. The purpose was to explore the factors influencing EAMs, tissue-specific/systemic EAMs, and the concurrence of several EAMs.

**Patients and methods:**

In total, 519 inpatients with RA were enrolled. The clinical characteristics, laboratory parameters, and medications of RA patients and the details of EAMs were recorded carefully. Differences between groups were examined by a Chi-square test, independent samples *t* test, one-way analysis of variance, Mann–Whitney *U* test, and Kruskal–Wallis *H* test. Binary and ordinal logistic regression analyses were performed to determine the relationship between influential factors and EAMs, tissue-specific/systemic EAMs, and the concurrence of several EAMs.

**Results:**

The morbidity of EAMs was 44.70%. Male sex, age, and traditional Chinese medicine (TCM) were independent influential factors of EAMs, and a number of statistically significant influence factors were found in the multivariate analysis of tissue-specific/systemic EAMs. Finally, age, CRP levels, number of RA-affected types of joints, and TCM were the factors that independently influenced the concurrence of several EAMs.

**Conclusion:**

Influential factors identified in this study could be recommended in clinical work, which is hopeful to decrease the morbidity and mortality of EAMs in RA.

## 1 Introduction

Rheumatoid arthritis (RA) is a systemic autoimmune disease characterized by persistent joint inflammation that affects approximately 1–2% of the population worldwide and occurs mainly in female patients who have higher susceptibility [1]. Although RA is primarily an articular disease, there are still a series of extra-articular manifestations (EAMs) associated with RA, such as ocular, pleuropulmonary, cardiovascular, renal, cutaneous, neurological system, and lymph node manifestations. EAMs are serious complications or outcomes of long-term systemic inflammation associated with increasing morbidity and mortality [1], and it was confirmed in a population-based study that the presence of EAMs was the strongest predictor of mortality in RA patients [2]. Nearly 40–50% of RA patients may suffer from some kind of EAM at the onset of disease or during the disease course [3,4], and the morbidity varies based on different study designs and inclusion criteria [5].

The aetiology and pathogenesis of EAMs are currently unclear. Cumulative studies have revealed that severe EAMs could be attributed to environmental factors, chronic systemic inflammation, and treatment [1,6,7]. Environmental factors, male sex, smoking habit, and dietary habits are thought to be related to EAMs [3]. Additionally, some innate or adaptive immune responses caused by complex interactions between genetic and environmental factors result in chronic systemic inflammation [6] and are characterized by increased levels of circulating immune complexes (CICs), low levels of complement factor 4 (C4), and higher counts of CD4+ T cells and B cells; besides, higher levels of rheumatoid factor (RF) were found in RA patients with EAMs than in those without EAMs, which suggests that RF may play a role in the formation of CICs and be involved in the persistence of systemic inflammation [8]. Thus, RA patients develop a higher risk of experiencing EAMs; nearly half of RA patients will suffer from EAMs due to a lack of recognizing the risk factors; those with risk factors should be screened on a regular basis to avoid the occurrence of EAMs and to allow specialists to provide interventions. With the improvement of RA treatment, the prognosis is improving among RA patients; nevertheless, it cannot be denied that medications have a certain impact on the occurrence of EAMs to some extent. A few case reports suggest a reduced incidence of severe EAMs in RA patients treated with cyclophosphamide, methotrexate (MTX), and high-dose corticosteroid treatment; however, the effect of tumour necrosis factor (TNF)-inhibitors on EAMs is still under debate [1,7].

Based on the background of EAMs in RA, we hypothesized that some of the clinical characteristics, laboratory parameters, and medications might be connected with EAMs. In this research, we collected general information on RA patients and aimed to explore the factors influencing EAMs and tissue-specific/systemic EAMs. The second purpose of the research was to screen the risk factors or the protective factors for patients suffering from a greater number of EAMs.

## 2 Patients and methods

**Informed consent:** Informed consent was obtained from all individuals included in this study.

**Ethics approval:** This retrospective study involving human participants was in accordance with the ethical standards of the institutional and national research committee and with the 1964 Helsinki Declaration and its later amendments or comparable ethical standards. The General Hospital of Western Theater Command approved this study.

### 2.1 Design and patients

The retrospective study was performed with 519 inpatients who were diagnosed with RA between January 2014 and August 2019 at the General Hospital of Western Theater Command (Chengdu, China). The inclusion criteria met the diagnostic criteria that satisfied the American College of Rheumatology 2010 revised criteria [9]. The exclusion criteria included (1) RA patients with incomplete clinical data, (2) RA patients with other autoimmune diseases, (3) RA patients who were pregnant, and (4) RA patients with tumours.

### 2.2 Influential factors

#### 2.2.1 Clinical characteristics

A series of common clinical characteristics were included, such as patient ID, sex, age, age at disease diagnosis, disease duration, smoking habit, and number of RA-affected types of joints.

#### 2.2.2 Laboratory parameters and a series of rheumatologic indicators

Erythrocyte sedimentation rate (ESR), C-reactive protein (CRP), RF, anti-cyclic citrullinated peptide antibody (ACPA), immunoglobulin G (IgG), immunoglobulin M (IgM), immunoglobulin A (IgA), complement 3 (C3), complement 4 (C4), and lactate dehydrogenase were recorded carefully.

#### 2.2.3 Medication

Treatment history of RA patients was recorded, such as hydroxychloroquine (HCQ), biological agents (e.g., tocilizumab, etanercept, infliximab, adalimumab, and golimumab), MTX, sulfasalazine (SAZ), leflunomide (LEF), and steroid hormones. The traditional Chinese medicine (TCM) included *Tripterygium wilfordii*, *Red paeonia*, *Astragalus membranaceus*, *Cassia* twig, prepared liquorice root, and *Ephedra* herb [10].

### 2.3 EAMs

The EAMs shown in Table 1 were selected according to the criteria reported by Prete et al. [11]. The observational outcome was identified as the presence of EAMs if there exists at least one EAM, and the specific tissue/systemic EAMs were diagnosed by specialists according to the corresponding diagnostic criteria.

**Table 1:** EAMs in RA

Organ/system involved in RA	EAMs observed
OMs	SICCA syndrome, keratoconjunctivitis sicca, scleritis, episcleritis
Pleuropulmonary involvement	Pleuritis, pleural effusion, interstitial lung disease, rheumatoid pulmonary nodules
Cardiovascular system	Pericarditis, myocarditis, valvular disease, arrhythmias, ischaemic heart disease, hyperlipidaemia, hyperglycaemia, hypertension
Renal involvement	Drug-induced renal injuries, secondary amyloidosis, mesangial glomerulonephritis, interstitial nephritis
Cutaneous involvement	Rheumatoid nodules, small-vessel vasculitis, splinter haemorrhages, digital gangrene, painful ulcers, Raynaud’s phenomenon
Neurological involvement	Entrapment neuropathy, mononeuritis multiplex, chronic inflammatory demyelinating polyneuropathy, carpal tunnel syndrome, peripheral neuropathy
LNE	Epitrochlear lymph nodes, axillary lymph nodes, mediastinal lymph nodes, hilar lymph nodes, inguinal lymph nodes

RA = rheumatoid arthritis, EAMs = extra-articular manifestations, OMs = ocular manifestations, LNE = lymph node enlargement.

### 2.4 Statistical analyses

All statistical analyses were performed with SPSS version 23.0 (IBM Corp, USA). Distributions of continuous variables were examined by the Kolmogorov–Smirnov test. The normal distribution continuous variables were presented using the mean values with standard deviations, and differences in continuous variables between two observed clinical outcomes (RA patients with EAMs vs RA patients without EAMs) were examined by independent-samples *t* test. One-way analysis of variance was used to compare the mean values between multiple groups (three ranked tissue-specific/systemic EAMs). We classified the number of affected tissue-specific/systemic EAMs into three categories: the lowest category indicates 0 tissue-specific/systemic EAMs, the middle one indicates 1 tissue-specific/systemic EAM, and the highest rank indicates 2–4 tissue-specific/systemic EAMs. The skewed distribution continuous variables were presented using the median (P_25_, P_75_), and the Mann–Whitney *U* test and Kruskal–Wallis *H* test were used to identify the differences in continuous variables between two groups (RA patients with EAMs vs RA patients without EAMs) and multiple groups (three ranked tissue-specific/systemic EAMs). Categorical variables were presented in frequencies/percentages, and distributions of categorical variables between observed clinical outcomes were estimated using cross-tabulations, and Chi-square tests were used to identify differences in categorical variables across the observed clinical outcomes. The associations of influential factors with EAMs and tissue-specific/systemic EAMs were explored using odds ratios (ORs) and 95% confidence intervals (CIs) in binary logistic regression analysis. Additionally, possible influential factors associated with a greater number of EAMs were screened by ordinal logistic regression analysis. Both the binary and ordinal logistic regression analyses have the function of adjusting confounding factors so that the final calculated influential factors are independent. All variables that were found to be significantly associated with tissue-specific/systemic EAMs using univariate analyses were included in binary and ordinal logistic regression analyses. *P* values ≤ 0.05 were considered statistically significant.

## 3 Results

### 3.1 Characteristics associated with EAMs

According to the inclusion criteria, 519 RA patients were included (232 RA patients with EAMs and 287 RA patients without EAMs). The morbidity of EAMs among RA patients was 44.70% (95% CI = 40.50–49.10). We compared clinical characteristics, laboratory parameters, and medications between two groups, and there were significant differences between two groups in terms of sex, age, age at disease diagnosis, smoking history, number of RA-affected types of joints, RF, and treatment with SAZ, biological agents, LEF, and TCM (Table S1).

### 3.2 Influential factors related to EAMs

In binary logistic regression analysis, through adjusting for the confounding factors (age at disease diagnosis, smoking history, RF, and treatment with biological agents, SAZ, and LEF), male sex (OR = 2.06, 95% CI = 1.36–3.32), age (OR = 1.03, 95% CI = 1.02–1.05), and treatment with TCM (OR = 0.59, 95% CI = 0.38–0.91) were independently associated with EAMs (Table 2).

**Table 2:** Binary logistic regression analysis of EAMs

	OR	95% CI	*P*-value
Sex (male vs female)	2.06	1.36–3.32	0.001
Age	1.03	1.02–1.05	<0.001
TCM (yes vs no)	0.59	0.38–0.91	0.017

OR = odds ratio; 95% CI = 95% confidence interval. The *P* value was obtained by binary logistic regression analysis. TCM = traditional Chinese medicine.

### 3.3 Characteristics associated with tissue-specific/systemic EAMs

#### 3.3.1 Ocular system

The morbidity of the ocular system was 3.85% (95% CI = 2.30–5.60). There were significant differences between two groups (RA patients with ocular involvement vs RA patients without ocular involvement) in terms of sex, CRP, ACPA, IgG, IgA, IgM, and treatment with HCQ (Tables S2–S4).

#### 3.3.2 Pleuropulmonary system

The morbidity of the pleuropulmonary system was 11.75% (95% CI = 8.90–14.60). There were significant differences between two groups (RA patients with pleuropulmonary involvement vs RA patients without pleuropulmonary involvement) in terms of sex, age, smoking history, RF, IgM, and treatment with biological agents, MTX, TCM, and steroid hormones (Tables S2–S4).

#### 3.3.3 Cardiovascular system

Among the 519 RA patients, the cardiovascular system was the system that was the most affected (27.55%, 95% CI = 23.50–31.60). There were significant differences between two groups (RA patients with cardiovascular involvement vs RA patients without cardiovascular involvement) in terms of sex, age, age at disease diagnosis, number of RA-affected types of joints, and treatment with TCM and LEF (Tables S2–S4).

#### 3.3.4 Renal system

The morbidity of the renal system was 1.54% (95% CI = 0.60–2.70). There were significant differences between two groups (RA patients with renal involvement vs RA patients without renal involvement) in terms of age, age at disease diagnosis, ESR, and IgA (Tables S2–S4).

#### 3.3.5 Cutaneous system

The morbidity of the cutaneous system was 2.50% (95% CI = 1.30–4.00). There were significant differences between two groups (RA patients with cutaneous involvement vs RA patients without cutaneous involvement) in terms of CRP and treatment with MTX (Tables S2–S4).

#### 3.3.6 Neurological system

The neurological system was the system that was the least affected (0.39%, 95% CI = 0.00–1.00). The results of neurological involvement could not be calculated because of the limited number of RA inpatients that were affected (Tables S2–S4).

#### 3.3.7 Lymph node enlargement (LNE)

The morbidity of LNE was 13.49% (95% CI = 10.40–16.40). There were significant differences between two groups (RA patients with LNE vs RA patients without LNE) in terms of sex, smoking history, CRP, ACPA, IgM, and treatment with biological agents, TCM, and SAZ (Tables S2–S4).

### 3.4 Influential factors of tissue-specific/systemic EAMs

Through binary logistic regression analysis, several significant influential factors associated with tissue-specific/systemic EAMs were found. In terms of ocular diseases, high levels of ACPA were found to be associated with greater susceptibility to ocular diseases (OR = 1.00, 95% CI = 1.00–1.00). Greater age (OR = 1.03, 95% CI = 1.01–1.06), smoking history (OR = 3.66, 95% CI = 1.87–7.16), high levels of IgM (OR = 1.44, 95% CI = 1.06–1.96), and history of treatment with MTX (OR = 0.40, 95% CI = 0.19–0.87), TCM (OR = 0.34, 95% CI = 0.17–0.66), and steroid hormones (OR = 3.24, 95% CI = 1.06–9.90) were independently associated with pleuropulmonary disorders. Male sex (OR = 1.67, 95% CI = 1.07–2.60), greater age (OR = 1.04, 95% CI = 1.03–1.06), and history of treatment with TCM (OR = 0.57, 95% CI = 0.36–0.91) were independently associated with cardiovascular involvement. High levels of CRP (OR = 1.01, 95% CI = 1.00–1.02) and history of treatment with MTX (OR = 0.15, 95% CI = 0.05–0.47) were independently associated with cutaneous diseases. Smoking history (OR = 2.55, 95% CI = 1.38–4.73), high levels of CRP (OR = 1.00, 95% CI = 1.00–1.01), ACPA (OR = 1.00, 95% CI = 1.00–1.00), and history of treatment with TCM (OR = 0.31, 95% CI = 0.17–0.55) were independently associated with LNE. However, there were no significant variables that were found to be associated with renal disorders (Table 3).

**Table 3:** Binary logistic regression analysis of tissue-specific/systemic EAMs

	OR	95% CI	*P*-value
OMs			
ACPA	1.00	1.00–1.00	0.007
Pleuropulmonary involvement			
Age	1.03	1.01–1.06	0.017
Smoking habit (yes vs no)	3.66	1.87–7.16	<0.001
IgM	1.44	1.06–1.96	0.020
MTX (yes vs no)	0.40	0.19–0.87	0.020
TCM (yes vs no)	0.34	0.17–0.66	0.002
Steroid hormones (yes vs no)	3.24	1.06–9.90	0.039
Cardiovascular system			
Sex (male vs female)	1.67	1.07–2.60	0.023
Age	1.04	1.03–1.06	<0.001
TCM (yes vs no)	0.57	0.36–0.91	0.017
Cutaneous involvement			
CRP	1.01	1.00–1.02	0.027
MTX (yes vs no)	0.15	0.05–0.47	0.001
LNE			
Smoking habit (yes vs no)	2.55	1.38–4.73	0.003
CRP	1.01	1.00–1.01	0.010
ACPA	1.00	1.00–1.00	0.004
TCM (yes vs no)	0.31	0.17–0.55	<0.001

OR = odds ratio; 95% CI = 95% confidence interval. The *P* value was obtained by binary logistic regression analysis. ACPA = anti-cyclic citrullinated peptide antibody, IgM = immunoglobulin M, CRP = C-reactive protein, MTX = methotrexate, TCM = traditional Chinese medicine, OMs = ocular manifestations, LNE = lymph node enlargement.

### 3.5 Influential factors associated with the concurrence of several EAMs

In an attempt to identify the influential factors associated with a greater number of EAMs, ordinal logistic regression analysis was performed. After adjusting for the confounding factors (age at disease diagnosis, RF, sex, smoking history, and treatment with HCQ, biological agents, MTX, SAZ, and LEF), age (OR = 1.04, 95% CI = 1.02–1.05), CRP levels (OR = 1.01, 95% CI = 1.00–1.01), number of RA-affected types of joints (OR = 0.72, 95% CI = 0.54–0.94), and treatment with TCM (OR = 0.42, 95% CI = 0.28–0.64) were found to be related to a greater number of EAMs (Table 4).

**Table 4:** Ordinal logistic regression analysis of the number of EAMs

	OR	95% CI	*P*-value
Age	1.04	1.02–1.05	<0.001
CRP	1.01	1.00–1.01	0.001
Number of RA-affected types of joints	0.72	0.54–0.94	0.017
TCM (yes vs no)	0.42	0.28–0.64	<0.001

OR = odds ratio, 95% CI = 95% confidence interval. *P* values were obtained by ordinal logistic regression analysis. CRP = C-reactive protein, TCM = traditional Chinese medicine.

## 4 Discussion

The EAMs of RA are characterized by multiple systemic manifestations and are usually associated with active and severe diseases, which doubles the risk of developing RA and increased mortality. To provide the possible options to prevent the occurrence of EAMs, our research screened the influential factors of EAMs as well as tissue-specific/systemic EAMs. Furthermore, we estimated the risk/protective factors affecting the occurrence of a higher number of EAMs.

Based on the inclusion and exclusion criteria, 519 RA patients were chosen. Among the population, EAMs were observed in 232 RA patients with a morbidity of 44.70%, which is slightly lower than that reported by Turesson et al. (50%) [12] but higher than those reported by Cimmino et al. (40%) [3] and Carmona et al. (36.2%) [13]. The discrepancy of the reported morbidity rates depends upon the research methodology employed and the differences in the population enrolled and is mainly due to a lack of consensus on the disease definitions of EAMs [5]. In terms of the influential factors screened, male sex and greater age were found to be the risk factors for EAMs in the binary logistic regression analysis. Similarly, it was reported by Turesson that severe EAMs tended to be more common in older age, and EAMs were also predicted using RA, ANA, smoking history, and early disability, but male sex was not found to be a strong predictor of EAMs [14]. The mechanisms underlying the association of male sex with EAMs are not understood clearly. The principles of treatment for RA primarily depend on comprehensive treatment, and it should be taken into consideration that pharmaceutical therapy can also have an impact on EAMs. In this study, we found that TCM treatment can protect RA patients from EAMs. A report based on a series of clinical and experimental studies indicated that Xinfeng capsules, a Chinese medicine, could significantly relieve the symptoms of EAMs [15]. Further study is needed to identify the associations between TCM and EAMs and the mechanisms underlying the impact of TCM on EAMs.

Ocular manifestations (OMs) are some of the EAMs and usually present as Sjögren’s syndrome. Other inflammatory ophthalmological conditions include keratoconjunctivitis sicca, scleritis, and episcleritis. All of these OMs are both sight- and life-threatening ones because of their association with systemic vasculitis [16]. A risk factor found in this study was ACPA, as reflected by another report that the presence of ACPA was significantly associated with OMs [17] and was the most prevalent biomarker among RA patients [18]. Therefore, ACPA can be both diagnostic and prognostic and can be easily detected before the occurrence of EAMs [19].

The overall morbidity rate of pleuropulmonary manifestations (PMs) in RA was 11.75%; pleuritis, interstitial lung disease, and pleural effusion were the most common symptoms in the present study. In general, the frequency of PM occurrence in RA is estimated to range from 4% to 68% [20]. The accurate frequency of PMs in RA is difficult to assess due to different disease definitions and techniques employed for diagnosis. In addition, the 10–20% mortality rate in RA is caused by PMs [21], second to cardiovascular manifestations [22]. On this basis, it is crucial to recognize any possible influential factors related to PMs, which will be instructive in future clinical work. In our study, we found that greater age, smoking habit, higher levels of IgM, and history of treatment with steroid hormones were considered risk factors of PMs, and treatments with MTX and TCM were considered protective factors of PMs. Other research reported that male sex, smoking habit, older age, longer disease duration, serum IgM, RF, ACPA, and ESR were associated with PMs in RA [23,24,25]. The PMs usually occur in RA patients with long-standing inflammation, particularly male smokers. The possible pathogenesis underlying this phenomenon is that smoking can produce a specific immune response to citrullinated proteins. On this basis, whether citrullination occurs in lung parenchyma or not is the key question to elucidate the role of smoking in PMs [26]. Contradictory results were obtained with regard to the influences of medications on PMs, especially the link between interstitial lung disease and MTX. Various views concerning the increased risk of PMs in RA patients treated with MTX [27], the protective impact of MTX [28], and even no relation between PMs and usage of MTX [29] have been reported. Beneficial impacts of MTX were shown in this research. In addition, TCM and steroid hormones showed protective and risk effects, respectively, on EAMs.

Cardiovascular manifestations (CMs) were the most common EAMs among RA patients in this study, with a morbidity of 27.55%. Epidemiological studies about the morbidity of CMs in RA patients are rare; however, a series of reports have revealed an increased risk of mortality of up to approximately 50% compared to that of the general population [30]. CMs are the leading causes of death in RA patients, and the widely accepted risk factors include hypertension, insulin resistance, and smoking [31]. In our research, male sex and greater age were regarded as risk factors, which is consistent with the view that the risk of CMs among people with older age, male sex, and older age at disease diagnosis is underestimated, as reported by Sunjoo Boo et al. [32]. Inflammation is widely accepted to be associated with an increased risk of CMs and is the hallmark of RA. Additionally, a series of pro-inflammatory molecules are involved in the pathogenesis of CMs, such as CRP, ESR, and cytokines [33,34]. These molecules not only participate in facilitating endothelial dysfunction and structural abnormalities of vessels but also induce changes in dyslipidaemia, insulin resistance, and oxidative stress [6]. In addition, a history of treatment with TCM was considered a protective factor of CMs in this study. A few medications, such as MTX and biological agents, were thought to decrease the risk of CMs [35], and our research suggests that TCM can protect RA patients from cardiovascular diseases, which would add to the limited research on medications due to the lack of TCM use abroad.

Cutaneous manifestations (CMs) can be present during the entire disease course, and a mass of CMs is an indication of disease severity and activity, with increased mortality and morbidity [36]. Rheumatoid nodules are the most common characteristics of CMs, and other CMs are also involved, including rheumatoid vasculitis, painful ulcers, and Raynaud’s phenomenon [36]. CMs often occur in RA patients who tested positive for RF and ACPA, who have a smoking history, and who were treated with TNF-α agents and LEF [14]. Additionally, higher levels of CRP were considered a risk factor, and treatment with MTX was considered a protective factor for CMs in this study. The results reported by numerous researchers are complementary for identifying the possible influential factors in cutaneous involvement.

Draining lymph nodes are responsible for immune surveillance, particularly generation of adaptive immune responses and maintenance of peripheral tolerance [37]. LNE in RA is generally recognized as an EAM of RA, with a frequency of 19–96% [37]. In our study, the incidence rate of LNE in RA was 13.49%, which is close to the ranges reported by other researchers. To investigate the influential factors related to developing LNE in RA patients, we found CRP and ACPA to be possible risk factors, which is consistent with the view reported by van Baarsen et al. that positivity for RA and/or ACPA was observed in early arthritis patients and in autoantibody-negative healthy controls [38]. More CD19+ B cells and activated CD69+ and CD8+ T cells were recognized in the lymph nodes of early RA patients and a trend of increasing CD19+ B cells was observed in high-risk subjects, suggesting that B cells play a role in the pathogenesis of LNE [39]. Additionally, smoking history was considered a risk factor, and a history of treatment with TCM was considered a protective factor associated with LNE. As far as we know, no previous reports have concluded the effects of smoking and TCM on RA patients with LNE; our study might, therefore, reveal the relationship between influential factors and LNE.

Severe EAMs often indicate the severity of the condition and are related to markedly elevated mortality in RA patients. To afford more efficient management for severe EAMs and possible predictors of a greater number of EAMs, we ran ordinal regression analysis and found that greater age and higher CRP levels were considered risk factors of the concurrence of several EAMs. Additionally, we discovered the phenomenon that RA patients with fewer types of RA-affected joints were more likely to suffer from severe EAMs, which may indicate that some RA patients present with EAMs as initial symptoms of RA, while the articular manifestations are less serious. The result may, to some extent, depend upon the population selected and the study setting; this is the first study to report the association between the types of RA-affected joints and the concurrence of several EAMs. Further research is needed to confirm the conclusion and explore the pathogenesis. Besides, a history of TCM treatment was found to decrease the risks of a greater number of EAMs. The addition of TCM to RA therapy can benefit RA treatment and prevent EAMs and the occurrence of a greater number of EAMs. We have found that TCM may be helpful in the prevention of all kinds of EAMs, whereas reports concerning associations between TCM and EAMs are rare. More research is needed to determine the relationship between TCM and EAMs.

Several limitations are present in this study. These limitations should be acknowledged, and some of them need to be improved in further research. First, our study is a retrospective study, and the casual relationship could not be ascertained because of the coexistence of exposure and outcome. Second, the conclusions would be more convincing with a larger sample. Third, the presence or absence of EAMs we observed would be the result of the comprehensive effect of therapy instead of the sole variable we set, so with regard to the influences of treatment on EAMs, more clinical trials and case studies are needed. The exploration of additional potential predictors and the confirmation of the suggested influential factors require further exploratory research.

## 5 Conclusions

In the present study, we found several potential influential factors that can be associated with EAMs, all kinds of EAMs, and even the concurrence of several EAMs, which provide directions for exploratory research to identify potential biomarkers of EAMs. Whereas research studies on EAMs are still lacking, reaching a consensus on the disease definitions of EAMs is urgent. Regular inspection and comprehensive analysis of influential factors are necessary to detect the presence of EAMs and initiate a treatment plan.
